# Differences in quantitative 99mTc-MIBI SPECT/CT parameters between parathyroid adenoma and hyperplasia in secondary hyperparathyroidism

**DOI:** 10.3389/fendo.2026.1883661

**Published:** 2026-07-15

**Authors:** Yuhua Wang, Ye Liu, Yahong Long, Na Li, Wanchun Zhang, Haiyan Liu

**Affiliations:** 1 Department of Nuclear Medicine, Third Hospital of Shanxi Medical University, Shanxi Bethune Hospital, Shanxi Academy of Medical Sciences, Tongji Shanxi Hospital, Taiyuan, China; 2 Department of Nuclear Medicine, First Hospital of Shanxi Medical University, Shanxi Medical University, Taiyuan, Shanxi, China

**Keywords:** parathyroid adenoma, parathyroid hyperplasia, secondary hyperparathyroidism, SPECT/CT, standardized uptake value

## Abstract

**Objective:**

The aim of this retrospective study was to investigate the differences in early- and delayed-phase maximum standardized uptake value (SUVmax) and lesion volume on ^99m^Tc-sestamibi (99mTc-MIBI) SPECT/CT between parathyroid adenoma (PA) and parathyroid hyperplasia (PH) in patients with secondary hyperparathyroidism (SHPT).

**Materials and methods:**

A total of 111 patients with stage 5 chronic kidney disease (CKD5) undergoing hemodialysis were enrolled in this study. All patients underwent preoperative ^99m^Tc-MIBI SPECT/CT scintigraphy for the detection and localization of parathyroid lesions. Quantitative SPECT/CT was performed after planar imaging, and the SUVmax and volume of each positive parathyroid lesions were measured on SPECT/CT. Linear mixed-effects models accounting for within-patient clustering were used to compare parameters between PA and PH, with further adjustment for lesion volume. Receiver operating characteristic (ROC) curve analysis was performed to evaluate the discriminative ability of these parameters.

**Results:**

After adjustment for within-patient clustering, PA lesions showed significantly higher early-phase SUVmax (*β* = 1.473, *P* < 0.001), delayed-phase SUVmax (*β* = 0.745, *P* < 0.001), and lesion volume (*β* = 0.571, *P* < 0.001) than PH lesions. After further adjustment for lesion volume, the difference in early-phase SUVmax remained significant (*β* = 0.542, *P* = 0.034), whereas the difference in delayed-phase SUVmax was no longer significant (*P* = 0.404). Lesion volume was strongly associated with both early- and delayed-phase SUVmax (both *P* < 0.001). A ROC curve analysis showed that the area under the curve (AUC) for early-phase SUVmax and lesion volume was 0.612 and 0.621, respectively, for distinguishing PA from PH.

**Conclusion:**

Lesion volume is a major determinant of ^99m^Tc-MIBI uptake in SHPT, while early-phase SUVmax remains independently different after adjustment for volume. When combined with clinical presentation and other examinations, early-phase SUVmax may provide complementary biological information for graft selection.

## Introduction

Secondary hyperparathyroidism (SHPT) is a common complication of advanced chronic kidney disease (CKD), caused by chronic hypocalcemia and/or hyperphosphatemia. It may increase the risk of cardiovascular disease, mineral and bone disorders, and hyporesponsive anemia. Treatment options for SHPT mainly include medical therapy, parathyroidectomy (PTx), and ablation (1, 2). Patients who do not respond to medical treatment require surgery, including subtotal PTx, total PTx with autotransplantation, or total PTx without autotransplantation. Among these approaches, total PTx with autotransplantation is generally considered the preferred treatment for SHPT because it is associated with fewer adverse effects (3). However, persistent or recurrent SHPT may still occur after total PTx and can result from ectopic glands, supernumerary glands, or overgrowth of residual parathyroid tissue or autografts (4–6).

A variable number and distribution of parathyroid glands are commonly observed in patients with SHPT (7). Therefore, accurate preoperative localization is particularly important. Ultrasound is widely used because of its convenience and cost-effectiveness. ^99m^Tc-sestamibi (MIBI) imaging also plays an important role in SHPT, especially in the detection of ectopic lesions. With the widespread use of SPECT/CT, the sensitivity and consistency of preoperative localization of parathyroid glands have improved considerably (8). Several studies involving quantitative SPECT/CT techniques (9–11) have applied standardized uptake value (SUV) measurements in ^99m^Tc-MIBI parathyroid imaging. One previous study (9) used SUV to differentiate adenomatous and hyperplastic parathyroid lesions in patients with primary hyperparathyroidism (PHPT). However, whether SUV can distinguish between adenomatous and hyperplastic parathyroid lesions in patients with SHPT has not yet been investigated. Therefore, this retrospective study aimed to investigate the differences in quantitative parameters on ^99m^Tc-MIBI SPECT/CT between parathyroid adenoma (PA) and parathyroid hyperplasia (PH) in patients with SHPT.

## Methods

### Clinical data

This retrospective study included 117 consecutive patients with stage 5 CKD who underwent parathyroidectomy for SHPT at our institution between February 2023 and December 2025. All patients underwent ^99m^Tc-MIBI SPECT/CT scintigraphy before surgery. Six patients were excluded because of non-quantitative SPECT/CT imaging. Therefore, data from 111 patients were included in the final analysis. The demographic and biochemical characteristics of the patients are shown in **Table 1**. The study was conducted in accordance with the Declaration of Helsinki and was approved by the Institutional Clinical Research Ethics Committee of Our Hospital (approval number: LYLL-2025-005/PJ79), which waived the requirement for individual informed consent because of the retrospective nature of the study.

**Table 1:** Demographic and clinical characteristics of 111 patients with SHPT.

Characteristic	Normal range	N	Median [interquartile range]
Gender *n (%)*			
Male		61*(54.95%)*	
Female		50 *(45.05%)*	
Age (years)		111	47 [37,56]
Dialysis (years)		111	10 [7,12]
Serum PTH (pg/mL)	12-88	111	
Preoperative			1518 [867.2.2, 2182]
Postoperative			32.10 [11.4, 64.6]
Serum P (mmol/L)	0.85-1.51	111	
Preoperative			2.01 [1.67, 2.36]
Postoperative			1.76 [1.46, 2.35]
Serum Ca (mmol/L)	2.20-2.65	111	
Preoperative			2.57 [2.39, 2.73]
Postoperative			2.40 [2.21, 2.69]

PTH, parathyroid hormone; P, phosphorus; Ca, calcium.

### ^99m^Tc-MIBI planar scintigraphy and SPECT/CT examinations

^99m^Tc-MIBI planar imaging was performed using a dual-head gamma-camera equipped with low-energy high-resolution collimators (Siemens Symbia Intevo Bold). Planar images of the neck and mediastinum were acquired at 15 min (early-phase) and 2 h (delayed-phase) after intravenous injection of 555 MBq (15 mCi) of ^99m^Tc-MIBI. The planar scans were acquired using a 256 × 256 matrix, with 500 k counts collected per position and a 20% energy window centered on the 140 keV photopeak. Regional SPECT/CT fusion imaging was performed after each dual-phase planar acquisition. SPECT images were acquired with an acquisition time of 20 s per frame, a 6° step angle, 60 total frames, a 256 × 256 matrix, and a zoom factor of 1. CT images were acquired with the tube voltage set at 100 kV, while the tube current was determined by automatic dose modulation with a reference current of 60 mAs (12).

### SPECT/CT imaging analysis

The reconstructed images were evaluated visually and quantitatively by two experienced nuclear medicine physicians who were blinded to both the surgical and pathological results. Positive parathyroid lesions were defined as focal uptake in the neck or mediastinum on SPECT imaging with a corresponding independent soft tissue mass on CT imaging, or as an independent soft tissue mass located behind the thyroid without uptake of ^99m^Tc-MIBI. Three-dimensional volumes of interest were placed over each positive lesion. The voxel-based activity concentrations were converted into SUVs using the xSPECT Quant application, which accounted for patient weight, injected activity, residual syringe activity after administration, and the time interval between injection and image acquisition (12). The SUVmax of each parathyroid lesion was recorded. To ensure the accuracy of SUV quantification in this study, phantom calibration studies, including point source sensitivity calibration and volume sensitivity calibration, were performed before clinical SUV analysis according to the manufacturer’s protocol. The dimensions of each parathyroid lesion were measured on SPECT/CT images. The length and width were obtained from the transverse section showing the largest cross-sectional area, while the height was measured on the coronal or sagittal plane. Lesion volume was calculated using the following equation: V=π/6×length (cm)×width (cm)×height (cm).

### Histopathological classification

Postoperative pathological reports of all resected parathyroid lesions were collected. Based on the pathological findings, lesions were classified into three categories: parathyroid adenoma (PA), parathyroid hyperplasia (PH), and parathyroid carcinoma (PC). According to the standard pathological criteria established at our institution, PA was diagnosed when a nodular lesion presented with a well-defined fibrous capsule or a compressed rim of normal parathyroid tissue, and lacked capsular or vascular invasion. PH was diagnosed when multiple glands showed diffuse or nodular proliferation without a normal rim. PC was diagnosed based on histopathological evidence of vascular invasion, perineural invasion, or invasion of adjacent structures. Lesions reported as “adenomatoid hyperplasia” or “hyperplasia with adenomatoid changes” were classified into the PH group. All classifications were based on the original pathological reports without central re-review.

### Statistical analysis

Statistical analyses were performed using SPSS and GraphPad Prism. Continuous variables were expressed as mean ± SD or median (interquartile range), and categorical variables were expressed as counts or percentages. To account for clustering effects, linear mixed-effects models with patient ID as a random intercept were used to compare quantitative parameters (early-phase SUVmax, delayed-phase SUVmax, and lesion volume) between subgroups. A multivariable linear mixed-effects model was used to assess whether SUVmax independently predicted PA after adjustment for lesion volume. Receiver operating characteristic (ROC) curve analysis was performed to determine the optimal cut-off values for SUVmax and lesion volume. Statistical significance was defined as *P* < 0.05.

## Results

### Study population

All 111 patients were on hemodialysis and underwent total PTx with autotransplantation. The characteristics of all patients are summarized in **Table 1**. Serum PTH and phosphorus levels were elevated preoperatively in all patients and decreased postoperatively. A total of 396 parathyroid lesions were surgically removed from 111 patients, of which 143 were pathologically confirmed as PA, 250 as PH, and 3 as PC (**Table 2**). The number of parathyroid lesions removed per patient was as follows: five lesions in two patients, four lesions in 77 patients, three lesions in 18 patients, two lesions in ten patients, and one lesion in four patients.

**Table 2:** Pathological classification of parathyroid lesions identified during surgery and by SPECT/CT imaging.

Modality	Pathological classification			Total
	Adenomas	Hyperplasia	Carcinoma	
Surgery	143 (36.11%)	250 (63.13%)	3 (0.76%)	396
SPECT/CT	136 (38.53%)	214 (60.62%)	3 (0.85%)	353

### ^99m^Tc-MIBI SPECT/CT image findings

All 111 patients underwent dual-phase ^99m^Tc-MIBI SPECT/CT imaging. A total of 357 lesions were detected by SPECT/CT, including 353 pathologically confirmed parathyroid lesions and four non-parathyroid lesions. Among the parathyroid lesions, there were 136 PAs, 214 PHs, and 3 PCs (**Table 2**). The number of parathyroid lesions detected per patient was five in one patient, four in 52 patients, three in 33 patients, two in 16 patients, and one in nine patients. Forty three parathyroid lesions (7 PAs and 36 PHs) were not detected by ^99m^Tc-MIBI imaging but were removed during surgery.

### Quantification of SPECT/CT

There were only three cases of PC, they were not included in the statistical analysis. The SUVmax values of these three PC lesions were 9.14, 16.67, and 6.09 on early-phase SPECT/CT and 8.10, 10.96, and 4.35 on delayed-phase SPECT/CT, while their volumes were 18.11, 2.18, and 0.51 cm^3^, respectively.

**Figure 1:**
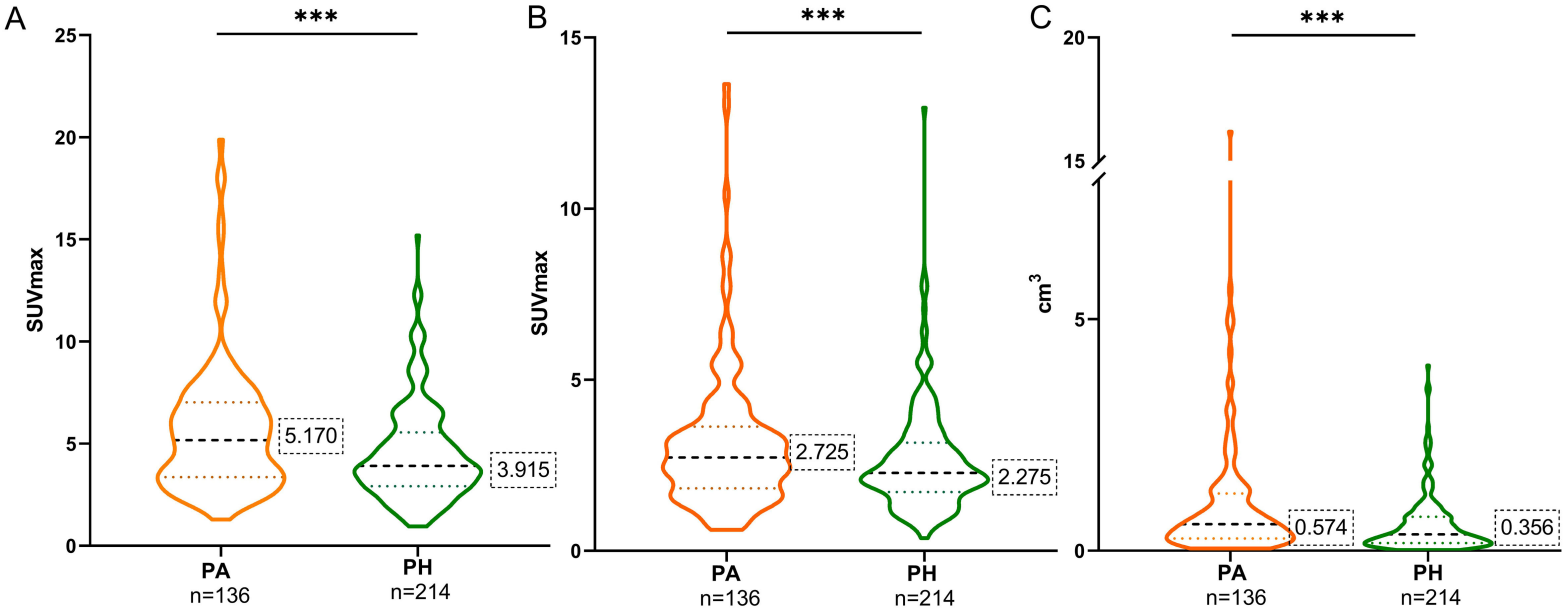
Violin plots comparing **(A)** early-phase SUVmax, **(B)** delayed-phase SUVmax, and **(C)** lesion volume between the PA and PH groups. Each violin represents the full data distribution without overlaid individual data points. Within each violin, the median is indicated by a thick dashed horizontal line, and the interquartile range (IQR) is delineated by thin dashed lines at the upper and lower quartiles. ***P < 0.001.

The median SUVmax values on early- and delayed-phase SPECT/CT for PA and PH, as well as the median lesion volume, are shown in **Figure 1**. Linear mixed-effects models accounting for the clustering of multiple lesions within the same patient were used to compare parameters between PA and PH. After adjustment for within-patient clustering, PA lesions showed significantly higher early-phase SUVmax (*β* = 1.473, 95% CI: 0.828-2.119, *P* < 0.001), delayed-phase SUVmax (*β* = 0.745, 95% CI: 0.329-1.161, *P* < 0.001), and lesion volume (*β* = 0.571, 95% CI: 0.299-0.842, *P* < 0.001) than PH lesions (**Figure 1**).

After further adjustment for lesion volume, the difference in early-phase SUVmax remained significant (*β* = 0.542, 95% CI: 0.043-1.041, *P* = 0.034). In contrast, the difference in delayed-phase SUVmax was no longer significant between PA and PH lesions (*β* = 0.135, 95% CI: -0.184-0.454, *P* = 0.404). Lesion volume was strongly and independently associated with both early-phase SUVmax (*β* = 1.561, 95% CI: 1.382-1.740, *P* < 0.001) and delayed-phase SUVmax (*β* = 1.011, 95% CI: 0.896-1.126, *P* < 0.001).

**Figure 2:**
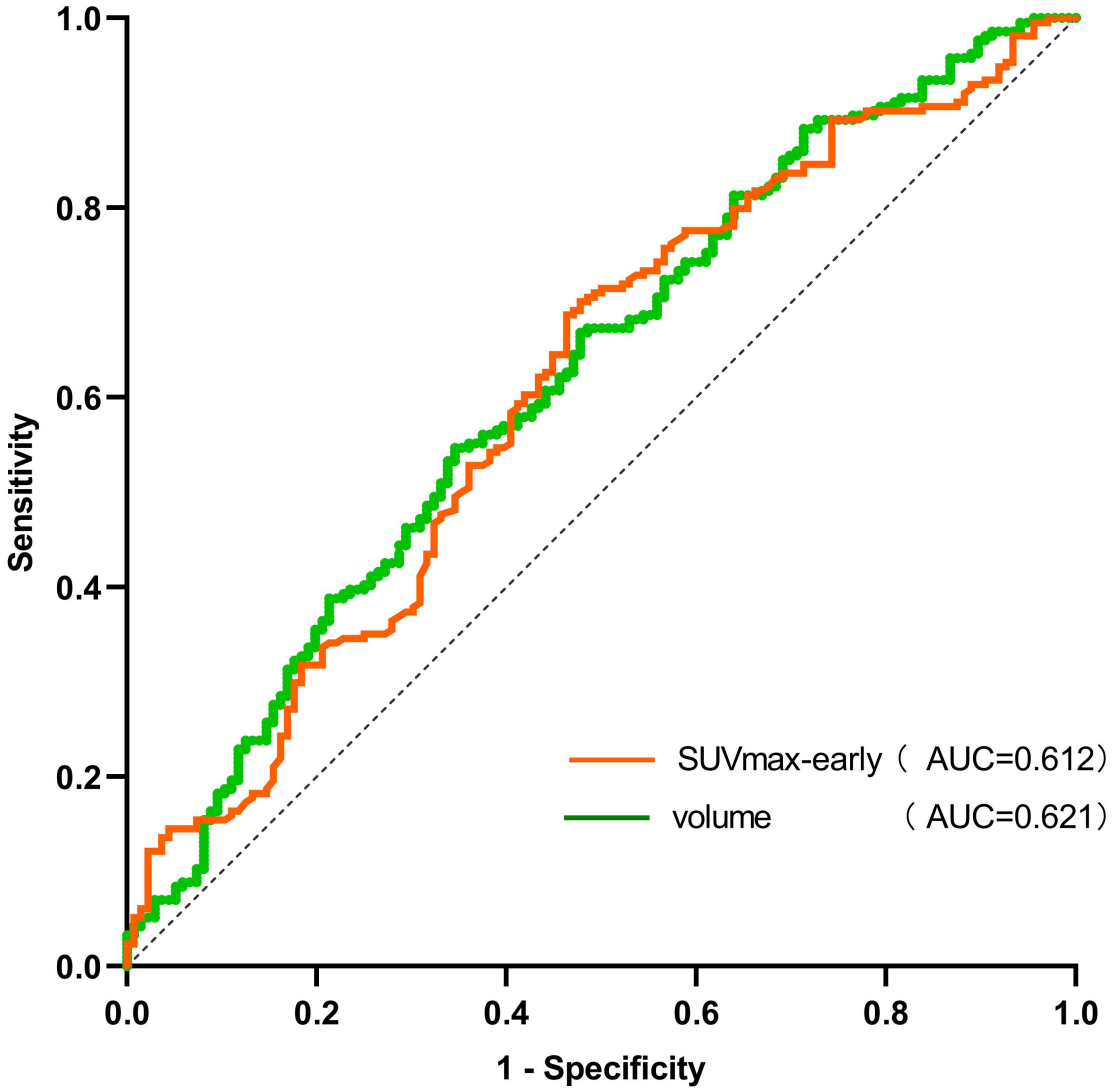
ROC curve analyses of early-phase SUVmax and lesion volume on SPECT/CT for differentiating PA from PH.

ROC curve analysis was performed to determine the optimal cut-off values for differentiating PA from PH. Early-phase SUVmax achieved an area under the curve (AUC) of 0.612 (95% CI: 0.552-0.673, *P* < 0.001), with a cut-off value of 4.925. Lesion volume showed an AUC of 0.621 (95% CI: 0.561-0.682, *P* < 0.001), with a cut-off value of 0.390 cm^3^ (**Figure 2**).

## Discussion

It has been reported that after 10 years of continuous dialysis, approximately 15% of patients with renal failure require PTx, and this proportion increases to 38% after 20 years of dialysis treatment (13). PTx combined with autologous transplantation is considered the optimal treatment for SHPT because it can effectively control hyperparathyroidism while avoiding permanent hypoparathyroidism (3). However, the underlying cause of SHPT cannot be eliminated, and recurrence may occur after PTx in the absence of kidney transplantation (14, 15). The recurrence rate may reach 10% (16). Persistent or recurrent SHPT often requires re-PTx (17), which increases the risk of recurrent laryngeal nerve (RLN) injury (18). Since the rate of re-PTx for persistent or recurrent SHPT is higher after total PTx with autotransplantation than after total PTx without autotransplantation (3), appropriate graft selection is particularly important. It is therefore recommended to select parathyroid glands with diffuse hyperplasia rather than nodular hyperplasia to reduce the risk of recurrence (19). Previous studies have shown that the highest risk of recurrence occurs in nodular lesions with a proliferative fraction greater than 1.5% (14). The progression of PH in SHPT is characterized by diffuse hyperplasia, early nodularity, nodular hyperplasia, and single nodules (20), eventually progressing to advanced-stage adenoma (21). Lesions with adenomatous features are thought to confer a higher recurrence risk than PH when used for autografting.

In our study, PA lesions showed significantly higher early-phase and delayed-phase SUVmax values, as well as larger volumes, than PH lesions after adjustment for within-patient clustering using linear mixed-effects models. These findings are consistent with those reported in a previous study of patients with primary hyperparathyroidism (9). Meanwhile, 83.7% of the lesions not detected by ^99m^Tc-MIBI imaging in our study were hyperplastic lesions, suggesting that ^99m^Tc-MIBI SUVmax is associated with the pathological characteristics of PA and PH. However, after further adjustment for lesion volume, the difference in early-phase SUVmax remained statistically significant but was markedly reduced, whereas the difference in delayed-phase SUVmax was no longer significant. These findings suggest that lesion volume is a major determinant of ^99m^Tc-MIBI uptake in SHPT, while early-phase SUVmax may reflect the intrinsic biological activity of parathyroid lesions independent of lesion size. This volume-independent functional information is in line with previous studies showing that ^99m^Tc-MIBI uptake reflects the functional status of hyperplastic parathyroid glands rather than simple gland enlargement (22). Higher ^99m^Tc-MIBI uptake grades have also been associated with the active growth phase of parathyroid lesions in SHPT. In addition, lower postoperative PTH levels have been reported when parathyroid tissue with the lowest ^99m^Tc-MIBI uptake ratio was selected for autografting (23). Our findings further suggest that this functional association is mainly reflected in early-phase imaging because early-phase SUVmax retained an independent difference after adjustment for lesion volume, whereas delayed-phase SUVmax did not. One possible explanation is the pharmacokinetic behavior of ^99m^Tc-MIBI. During the early phase, tracer uptake is likely influenced more by blood perfusion, and PA lesions usually have a rich blood supply (24). This perfusion-related difference may persist even after accounting for lesion size, potentially explaining why early-phase SUVmax retained independent discriminative value. In contrast, PA and PH lesions may not differ substantially in factors related to MIBI washout during the delayed phase, which may explain the loss of independent discriminatory value. Therefore, early-phase imaging may provide greater value than delayed-phase imaging when SUVmax is used to distinguish parathyroid adenoma from hyperplasia.

Several studies have examined the clinical significance of parathyroid lesion size. The size of PTGs identified during surgery or by ultrasound can help distinguish nodular hyperplasia from diffuse hyperplasia (21, 25). Using PTGs with a maximum diameter of less than 14 mm as autografts during surgery may reduce the risk of recurrent SHPT (6), while a total parathyroid volume greater than 2.65 cm^3^ has been reported to predict postoperative recurrence (26). Consistent with these findings, our study showed that PA lesions had significantly larger volumes than PH lesions. ROC curve analysis showed that the AUCs of early-phase SUVmax and lesion volume were both below 0.7, indicating limited ability to distinguish PA from PH at the individual level. These findings suggest that neither parameter can serve as an independent and reliable discriminator. We speculate that several factors may contribute to this result. First, hyperplastic lesions account for a large proportion of lesions in patients with SHPT, and adenoma and hyperplasia may represent a pathological continuum rather than completely separate entities. Second, partial volume effects may affect SUV measurements, particularly in smaller lesions. Therefore, the diagnostic performance of a single parameter may be limited, and a multiparametric approach combining additional imaging findings and clinical features may be needed to improve differentiation between PA and PH in clinical practice.

High levels of PTH and serum phosphorus may contribute to the malignant transformation of parathyroid lesions (27). A review of the literature identified 36 cases of parathyroid carcinoma in hemodialysis patients, suggesting that higher PTH levels, larger lesion volume, and hypercalcemia may be associated with PC (28). In our study, all three PC lesions showed higher dual-phase SUVmax values than the median values observed in both the PA and PH groups, and two of them had larger lesion volumes. These findings suggest that markedly elevated ^99m^Tc-MIBI SUVmax may serve as a potential imaging marker for PC. They also support our strategy of selecting low-SUVmax tissue for autografting, as lesions with high SUVmax may be less suitable as graft tissue. Given the very small number of PC cases, this observation should be regarded as hypothesis-generating and requires further investigation.

Our study has several limitations. First, this was a retrospective study, and follow-up data were not available to evaluate the relationship between SUVmax and postoperative recurrence. Second, the AUC values were insufficient for individual-level clinical discrimination. Third, we did not apply recovery coefficient measurements and reproducibility assessments, which may have affected SUV accuracy in smaller lesions. Four, the number of PC cases was too small to allow meaningful statistical comparisons with PA or PH. Future prospective studies with larger sample sizes and longer follow-up periods are needed to validate the prognostic value of early-phase SUVmax for recurrence after autotransplantation and to explore multiparametric approaches that may improve predictive performance.

## Conclusion

Lesion volume is a major determinant of ^99m^Tc-MIBI uptake in SHPT, while early-phase SUVmax retains an independent difference after adjustment for lesion volume. When combined with clinical presentation and other examinations, early-phase SUVmax may provide complementary biological information for graft selection.

## Data Availability

The datasets generated and analyzed during this study are not publicly available because they contain patient-specific clinical information protected under patient confidentiality and privacy regulations. Access to the data is restricted to the research team and can only be granted to external researchers upon reasonable request to the corresponding authors, subject to approval from the institutional ethics committee and a signed data use agreement. Requests to access these datasets should be directed to zhangwanchun@sxbqeh.com.cn; liuhaiyan@sxmu.edu.cn.
